# LiDAR-360 RGB Camera-360 Thermal Camera Targetless Calibration for Dynamic Situations

**DOI:** 10.3390/s24227199

**Published:** 2024-11-10

**Authors:** Khanh Bao Tran, Alexander Carballo, Kazuya Takeda

**Affiliations:** 1 Graduate School of Informatics, Nagoya University, Furo-cho, Chikusa-ku, Nagoya 464-8601, Japan; takeda@g.sp.m.is.nagoya-u.ac.jp; 2 Faculty of Engineering and Graduate School of Engineering, Gifu University, 1-1 Yanagido, Gifu City 501-1193, Japan; alex@gifu-u.ac.jp; 3 Institutes of Innovation for Future Society, Nagoya University, Furo-cho, Chikusa-ku, Nagoya 464-8601, Japan; 4 Tier IV Inc., Nagoya University Open Innovation Center, 1-3, Meieki 1-chome, Nakamura-Ward, Nagoya 450-6610, Japan

**Keywords:** LiDARs, panoramic RGB cameras, panoramic thermal cameras, targetless calibration, ego-motion compensation

## Abstract

Integrating multiple types of sensors into autonomous systems, such as cars and robots, has become a widely adopted approach in modern technology. Among these sensors, RGB cameras, thermal cameras, and LiDAR are particularly valued for their ability to provide comprehensive environmental data. However, despite their advantages, current research primarily focuses on the one or combination of two sensors at a time. The full potential of utilizing all three sensors is often neglected. One key challenge is the ego-motion compensation of data in dynamic situations, which results from the rotational nature of the LiDAR sensor, and the blind spots of standard cameras due to their limited field of view. To resolve this problem, this paper proposes a novel method for the simultaneous registration of LiDAR, panoramic RGB cameras, and panoramic thermal cameras in dynamic environments without the need for calibration targets. Initially, essential features from RGB images, thermal data, and LiDAR point clouds are extracted through a novel method, designed to capture significant raw data characteristics. These extracted features then serve as a foundation for ego-motion compensation, optimizing the initial dataset. Subsequently, the raw features can be further refined to enhance calibration accuracy, achieving more precise alignment results. The results of the paper demonstrate the effectiveness of this approach in enhancing multiple sensor calibration compared to other ways. In the case of a high speed of around 9 m/s, some situations can improve the accuracy about 30 percent higher for LiDAR and Camera calibration. The proposed method has the potential to significantly improve the reliability and accuracy of autonomous systems in real-world scenarios, particularly under challenging environmental conditions.

## 1. Introduction

Combining multiple sensors is an effective method for various applications [1,2,3], as using a single sensor can be limited by its disadvantages. For example, although RGB cameras can provide detailed color imagery, facilitating the clear identification of objects and environments, these sensors can be impacted by adverse weather conditions, such as rain or nighttime. In contrast, thermal cameras can detect surface temperatures, enabling autonomous devices to operate effectively under low-light conditions or to identify concealed objects. However, elements like transparency or reflectivity can lead to inaccurate results. On the other hand, while LiDARs can accurately measure distances and create detailed three-dimensional maps, object detection tasks may be affected by the sparsity of the point cloud data. Fusing multiple sensors allows systems to maximize the advantages of each sensor type, enabling them to operate effectively in different environments, at various times, and across different distances [4]. To perform effective sensor fusion, calibration is a crucial step, ensuring that data from different sensors are accurately aligned. Calibration adjusts the parameters, allowing their outputs to be consistent for successful integration.

There are two common approaches to sensor calibration, which are target calibration [5,6] and targetless calibration [7,8,9,10]. In calibration, a target [11,12] is typically a known object or pattern used as a reference to calibrate sensors by providing fixed, measurable points in space. The target allows the system to accurately determine the relationship between sensors, such as their position and orientation relative to each other. In general, since the number of extracted features and distances can vary based on the characteristics of the sensors, target calibration tends to be more accurate  [13,14] than targetless calibration. Although most registration research focuses on using targets due to their considerable benefits, these methods require specific equipment conditions for calibration [15,16,17,18]. In contrast, calibration without specific targets can address this issue because these methods use information and features from random objects around the sensors. Additionally, using cameras with wide viewing angles [19,20,21] in combination with LiDARs has an even more positive impact than combining standard cameras with narrow viewing angles with LiDARs. Firstly, the ability to capture a 360-degree view provides better context for viewers and users. Besides that, these cameras can capture more detail across wide areas, which is useful for applications such as environmental monitoring. However, with mechanical LiDARs, information is collected from left to right or vice versa, leading to distortion in the captured shape in situations sensors moving with high speeds, which can have negative impacts on performances for other deployments, such as detection or mapping. Even in static situations, this phenomenon can occur if environmental changes, such as dust or smoke, cause data discrepancies. However, this distortion does not have as significant an impact as it does when devices are in dynamic situations.

**Figure 1:**
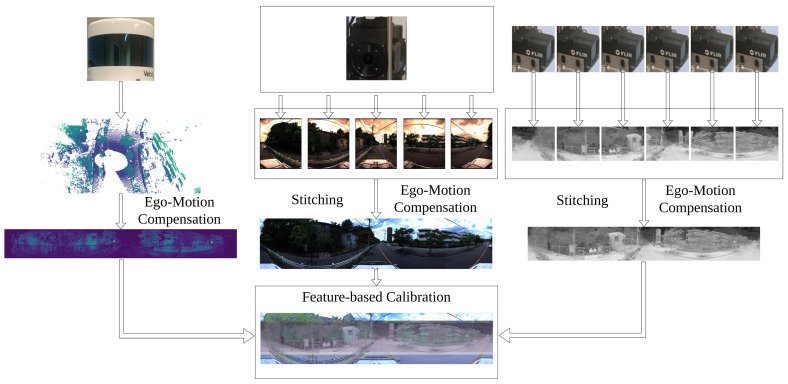
Visualization of the system including RGB cameras, thermal cameras, and LiDAR. 360 RGB camera and 360 thermal camera are made from independent cameras to remove blind spots. Images and point clouds are compensated to decrease negative impacts of motion. Then, point clouds and images are used for sensor calibration based on extracted features.

Figure 1 shows our system in this paper. Generally, we integrate independent cameras to form two panoramic cameras, effectively eliminating blind spots. After that, we enhance sensor calibration by removing the effects of ego-motion compensation across LiDARs, RGB cameras, and thermal cameras, which significantly improves the accuracy of targetless calibration methods. To achieve this, we estimate velocity from the LiDAR data, and use this information to correct the distortion in the sensor data, ensuring more precise and reliable measurements. The contribution of this paper are listed below:

1. Ensuring effective sensor operation in dynamic conditions requires addressing deformation correction caused by velocity influences, a critical task for reliable data acquisition [22]. Most current approaches leverage additional support devices [23] to re-measure speed and distance; however, while effective in ideal conditions, these devices can be significantly impacted by adverse weather or complex, obstacle-rich environments. Moreover, employing multiple devices increases computational demand, potentially introducing delays in real-world applications. To mitigate these limitations, we propose a novel way for calculating velocities from point clouds based on extracted key points from projected images.
2. Sensor calibration is always the first task performed to optimize systems. However, as mentioned above, target-specific calibration is not always feasible, particularly for 360-degree cameras. Additionally, widely used calibration methods with pre-existing datasets are typically suited to standard images, not 360-degree images, which are central to this paper. Therefore, we introduce a new method of targetless calibration in dynamic situations for 360 LiDARs and 360 RGB cameras based on the above approach of points accumulation.
3. The significant aspect ratio disparity between 360 and degree images and standard images can render conventional outlier removal and matching methods prone to overfitting. To improve the pairing efficiency of 360 images and point cloud datasets, we propose an advanced approach to enhance the percentage of matching features for 360 images and filter noise in pair features.
4. The code and dataset can be accessed at https://github.com/baokhanhtran/Multimodal-Targetless-Calibration (accessed on 6 October 2024).

This paper is structured as follows: Section 2 provides a comprehensive review of the state of the art related to calibration methods, with emphasis in targetless calibration. Section 3 presents our targetless calibration method for three modalities of 360 sensors, while Section 4 discusses our approach for ego-motion compensation for LiDAR and cameras. Section 5 corresponds to the analysis of our experimental results, while Section 6 provides important discussions and lessons learned. Section 7 concludes this paper.

## 2. Related Works

In this section, we will provide a comprehensive summary of the key issues addressed in this paper, including the calibration methods employed and the techniques used to mitigate distortion caused by ego-motion. The calibration approaches discussed encompass both target-based and targetless methods, each playing a distinct role in the system’s development. The target-based calibration, for instance, is applied to align and stitch multiple cameras together, resulting in the creation of two panoramic cameras that effectively eliminate blind spots. On the other hand, targetless calibration techniques are utilized to fine-tune the sensors, allowing for seamless operation without the need for predefined reference points. This dual approach not only optimizes the system’s overall calibration, but also improves its adaptability and precision in dynamic environments.

### 2.1. Calibration

#### 2.1.1. Target Calibration

**Figure 2:**
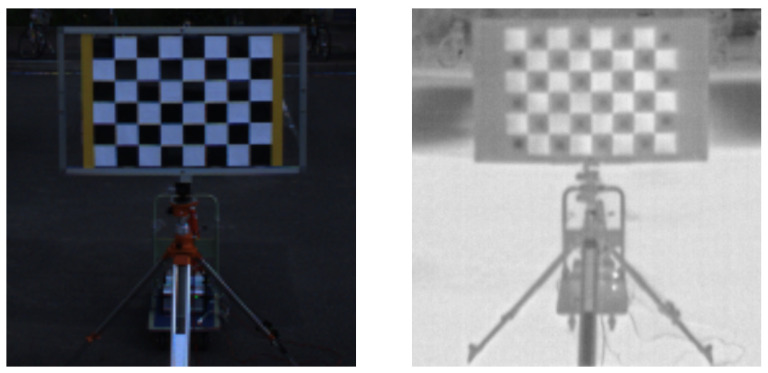
Visualization of the target detected by two types of cameras. The (**left image**) is the target detected by the RGB camera and the (**right image**) is the target detected by the thermal camera.

Target based methods such as the Autoware package  [24] are popular approaches to determining the relationships between different types of sensors. Targets are usually placed at known locations and angles within the workspace of the machine or equipment system. Common calibration targets are checkerboard patterns [25] with precisely known dimensions, which help in aligning and adjusting the sensor data. In addition to conventional checkerboards, specially designed patterns that incorporate a variety of shapes and materials  [26] are also employed to improve the detection capabilities of different sensors. In this paper, we use target calibration with a specially designed target [27] as Figure 2 to make ground truth for 360 RGB camera and 360 thermal camera dataset. This target is designed to control temperature.

Generally, target-based calibration achieves higher accuracy than targetless methods, as the presence of a designated target aids the sensor in accurately recognizing and aligning with calibration points. However, such methods often necessitate specialized equipment and controlled conditions. For instance, both the $O^{3}$ Calibration [28] and LVT2 Calibration [29] methods must be conducted in a laboratory setting with custom-designed targets tailored for precise calibration tasks. Conversely, targetless calibration offers advantages in flexibility and speed, facilitating quicker and more adaptable calibration without the need for specialized environments or equipment.

#### 2.1.2. Targetless Calibration

This is a method to perform calibration without special targets or objects. Instead of relying on targets, algorithms calculate necessary parameters based on data collected from sensors, physical systems, or special points in workspaces. In this paper, we will extract features on three types of sensors and then perform calibration. This method is called the feature-based method. LiDAR collects 3D point clouds, while cameras capture images with overlapping fields of view. Features including edges, planes, and angles are extracted from both LiDAR and camera data. Calibration parameters are then estimated to minimize differences between LiDAR points projected onto camera images and detected image features or vice versa. Calibration procedure is validated by projecting LiDAR points onto camera images and calculating loss. The process can be repeated with additional data to fine-tune parameters, ensuring high accuracy of sensor systems.

In recent years, the advantages of speed and convenience have increased interest in targetless calibration methods. However, most existing research [30,31,32] focuses on standard cameras, including RGB and thermal cameras, rather than 360-degree cameras. As a result, the full 360-degree scanning capability of mechanical LiDAR is often underutilized in detection and tracking applications, as blind spots remain in standard camera setups. Applying calibration methods developed for standard cameras to 360-degree cameras can lead to suboptimal outcomes due to distortions caused by the stitching process, which alters image size and shape. This highlights the need for developing a specialized and effective calibration method tailored to 360-degree sensor systems.

### 2.2. Ego-Motion Compensation

In real-world dynamic scenarios, movements of devices or vehicles can be classified into two primary categories, which are static and dynamic states. Static states of an object refers to a condition in which objects remain stationary relative to a specific reference frame over time. In this state, objects exhibit neither translational nor rotational motions, resulting in zero velocity at all points. Dynamic states of a vehicle encompass various scenarios, including forward motions, reverse motions, and turning motions. When a device is in a static state, geometry of scanned objects by LiDARs and captured by cameras is unchanged. Although certain environmental conditions, such as variations in reflectivity, may introduce slight alterations, these changes are generally negligible. In contrast, during dynamic conditions, extent of image distortion is contingent upon direction, speed, and acceleration of motions. While rapid rotational speed of modern LiDAR minimizes differences in distortion at low speeds, significant alterations in both position and shape become evident as devices operate at medium to high velocities. Distortions in point clouds and images caused by moving objects present challenges for applications such as calibration, detection, and feature extraction. Correcting these distortions is crucial for providing accurate representations of objects or areas [22,33]. A commonly used approach to ego-motion compensation involves estimating velocity and time.

There are two primary methods for estimating speed prior to applying distortion correction. The first method involves utilizing position or distance-determining devices such as Inertial Measurement Units (IMUs) and Global Navigation Satellite Systems (GNSS). However, these devices have certain limitations when combined with LiDARs. For example, IMU are prone to drift due to accumulated errors over time which reduces the accuracy of velocity estimates. GNSS also presents its own set of problems, such as signal interference. Latency in position updates can also impact precisions of velocity estimation. Additionally, in environments with weak GNSS signals such as indoors, underground, or in canyons, estimating velocity becomes more difficult. Several studies [23,34] have utilized IMU and GNSS, often incorporating algorithms like Kalman Filter to reduce noise in features.

This is the reason for developing a second method to estimate velocity based on LiDAR point clouds [35,36]. By using timestamps and coordinates of points within LiDAR point clouds, distance and velocity information can be extracted and calculated. This information is then used to address issues related to distortion correction. In this way, data are collected from a single type of sensor, minimizing risks of interference or information loss due to external factors.

### 2.3. Velocity Estimation

Velocity calculation is a necessary step in the ego-motion compensation problem. From the estimated velocity, a more accurate position of the object in the point clouds and images can be calculated. To calculate the velocity of the system, a commonly used method is to accumulate the point clouds to find the distance traveled. From the distance traveled and the timestamp from the LiDARs, the velocity of the system can be calculated.

Point cloud accumulation can be approached through three main methodologies. The first way involves utilizing devices such as GNSS or IMU to enhance functionalities of LiDAR systems [37,38]. In research, positional and orientation data from IMU and GNSS are used to accurately position and rotate each frame of point clouds. However, using IMU or GNSS can have some negatives as presented above.

The second approach involves adaptations of feature detection algorithms, originally designed for images, to point clouds, or conversions of point clouds into images for key point detection  [39,40]. This method mitigates computational burdens associated with processing 3D data and leverages advancements in image processing techniques.

The third method directly utilizes 3D point cloud data [41,42,43]. A common technique within this approach involves employing geometric registration algorithms to align and match point clouds, thereby deriving optimal transformation matrices between them. The principal advantages of this method are its simplicity and broad applicability across diverse types of point clouds and varying environmental contexts.

## 3. Targetless Calibration

In this section, we present our targetless calibration method applicable across all three sensor types, detailing the specific sensors used and describing the transformation process from standard to 360-degree cameras.

**Figure 3:**
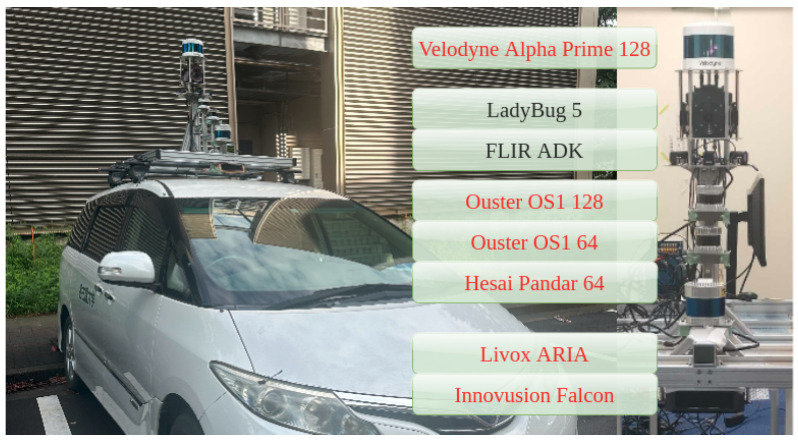
Our system includes sensors: LiDAR Velodyne Alpha Prime, LadyBug-5 camera, 6 FLIR ADK cameras, LiDAR Ouster-128, LiDAR Ouster-64 and LiDAR Hesai Pandar.

**Figure 4:**
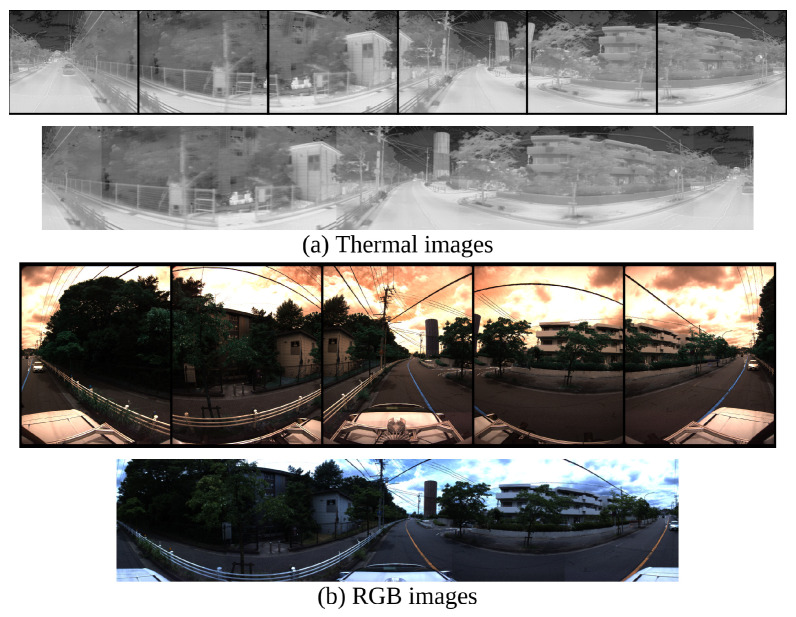
Visualization of stitching 360 thermal images and 360 RGB images.

In this paper, we use LiDAR Ouster-128, LiDAR Velodyne Alpha Prime [44], six FLIR ADK cameras [45] and LadyBug camera 5 [46], as in Figure 3, to record dataset and evaluate results. The LadyBug camera integrates multiple individual cameras and FLIR ADK cameras are arranged on a unified structure to capture comprehensive 360 RGB images and comprehensive 360 thermal images. The system is capable of recording extensive image data from all directions, thereby significantly enhancing the capacity to observe and analyze the surrounding environment. We stitch cameras to obtain a 360 RGB camera and 360 thermal images, as in Figure 4.

**Figure 5:**
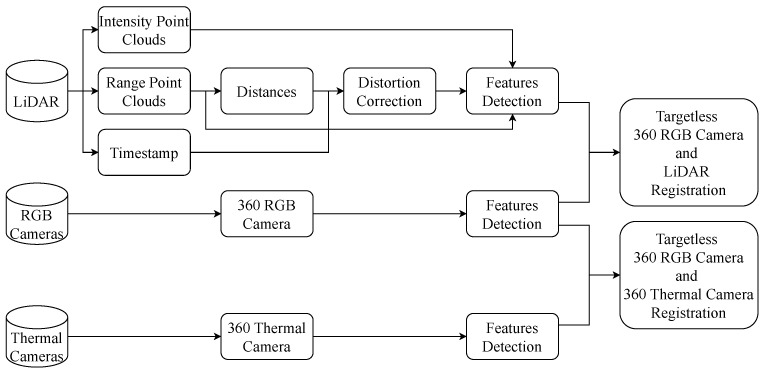
Pipeline of the registration process. The approach is divided into two parts, one part focuses on detecting key points from RGB images and thermal images, while the other part detects key points from images converted from LiDAR point clouds. For images generated from LiDAR point clouds, a velocity estimation step is required to perform distortion correction, ensuring the accurate positioning of the scanned points. After getting results from distortion correction, external parameters of LiDAR, 360 RGB camera and 360 thermal camera can be calibrated.

Our calibration process is outlined in Figure 5. Initially, data from RGB cameras, thermal cameras and LiDARs are input. For LiDAR, the intensity, range channels, and timestamp information are used. The range data of point clouds is utilized to estimate distances between frames using point cloud registration. From the estimated distance and timestamp of the LiDAR, distortion correction of the entire system can be calculated. Dynamic distortion correction is then applied to frames. From the applied new frames, key points of the LiDAR images are extracted. Related to cameras, the five RGB cameras and six thermal cameras are first stitched together to create a 360 RGB camera and a 360 thermal camera. Then, the key features from LiDAR images, 360 RGB images and 360 thermal images can be extracted for calibration.

### 3.1. Feature Extraction

#### 3.1.1. Feature Extraction from Camera Images

**Figure 6:**
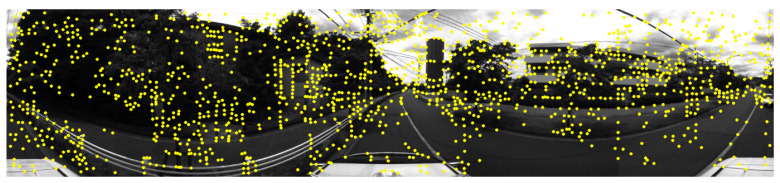
Visualization of features extracted from RGB images.

To extract possible features in images, we use SuperPoint [47]. SuperPoint is a deep learning algorithm used for keypoint detection and descriptor extraction in computer vision tasks such as feature matching. The algorithm identifies important points in images such as corners or edges, and then extracts a feature vector for each keypoint. These descriptors enable comparison and matching of points across different images. SuperPoint is trained using a self-supervised approach, allowing it to learn how to detect keypoints and descriptors from unlabeled data during the training process. Although maximizing the number of extracted points, noise in the frames will affect the point matching results between sensors. To decrease noise features, as in Figure 6, we present a method depending on the number of features in consecutive images.

**Figure 7:**
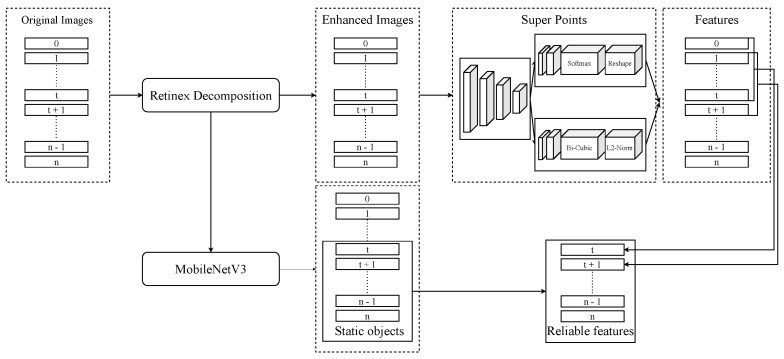
Pipeline of our approach. The first step is enhancing images by Retinex Decomposition. The second step is to extract key features from n+1 consecutive images. The third step is using MobileNetV3 to remove noise features on moving objects.

The method to detect features on RGB images is presented in Figure 7. Firstly, images are enhanced with Retinex Decomposition [48]. Retinex Decomposition is an image enhancement algorithm that aims to perceive consistent colors under varying lighting conditions. It decomposes an image into two components, which are reflectance and illumination. By separating these two components, Retinex can enhance the visibility of details in poorly lit areas while maintaining natural color appearance, making it useful for tasks such as low-light image enhancement and color correction. The algorithm is widely applied in image processing to improve visual quality under non-uniform lighting conditions.

**Figure 8:**
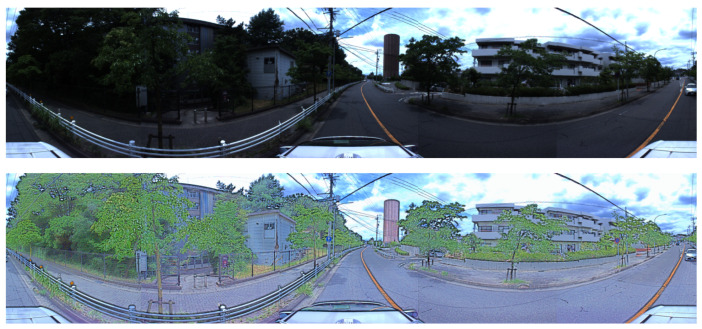
The (**above image**) shows results before being enhanced by Retinex Decomposition. The (**below image**) shows results after being enhanced by Retinex Decomposition.

By enhancing, as in Figure 8, boundaries between objects and the edges of objects can be more clearly emphasized, allowing for sharper distinctions between different regions. Additionally, visibility can be significantly enhanced, making details that were previously obscured more discernible. Moreover, the algorithm can reduce the impact of unwanted color casts, ensuring that colors appear more natural and accurate, even under challenging lighting conditions, to improve the overall clarity of the image.

**Figure 9:**
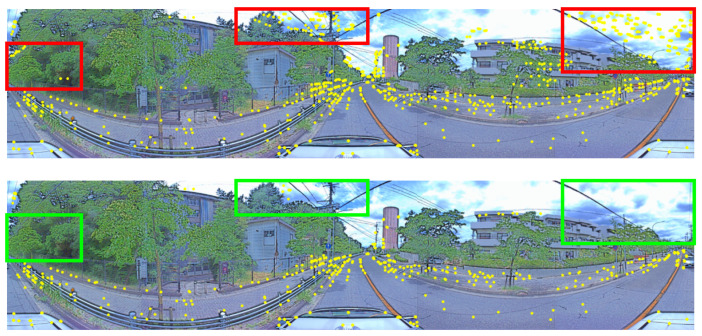
The (**above image**) including the red rectangles shows reliable features extracted from n+1 consecutive RGB images. The (**below image**) including the green rectangles shows reliable features after filtering by MobileNetV3.

In the second step, we use consecutive images n,n−1,…,1,0 to obtain reliable features in image *n*. Reliable features which are extracted by SuperPoint [47] are points that appear in all images. We use Brute-Force matchers [49] to evaluate with descriptors to find that features in frame *n* are also in frame n−1,…,0. The Brute-Force matcher is a straightforward algorithm used to match features between two frames in computer vision tasks. It works by comparing each feature’s descriptor from one frame to the descriptors of all features in other frames. After extracting features from both frames using SuperPoint, the matcher calculates the distance between every pair of descriptors. The feature in the first frame is then matched to the feature in the second frame with the closest descriptor, often the one with the smallest distance. The final step is removing unreliable features in moving objects by semantic segmentation. In this paper, we use MobileNetV3 [50]. This is a lightweight deep learning model designed to perform pixel-level classification tasks efficiently. In the context of semantic segmentation, MobileNetV3 serves as the backbone for extracting features from images. To handle segmentation tasks, MobileNetV3 is often paired with a segmentation head, which refines the feature maps and produces detailed segmentation masks. This combination provides an efficient solution for applications, where computational resources are constrained. These features in moving objects such as cars are likely to change orientation and position in different frames, and thus are not considered reliable points. Figure 9 is the result when applying segmentation masks on reliable features from consecutive images.

The algorithm is presented in Algorithm 1. In this algorithm, images captured by the panoramic camera serve as the input. These images are first enhanced using Retinex Decomposition to improve visibility and highlight important details. SuperPoint is then applied to extract key features from the enhanced images. To ensure reliability, the extracted features are filtered by Semantic Segmentation, which identifies and removes features located in unreliable regions, denoted as $U_{n}$. The remaining reliable features are grouped into the $F_{i}$ set. The Brute-Force matcher is then used to compare these features across multiple images, determining whether they represent the same points. If a feature consistently appears across all the required images, it is classified as a reliable feature.
**Algorithm 1** Feature extraction in RGB imagesINPUT    **Images** $I_{0},I_{1},\ldots ,I_{n}$RESULTS    **Reliable Features** R←{∅}    **Empty Dictionary** D←{∅}    **Unreliable Features** $U_{n}\leftarrow Segmentation\left(I_{n}\right)$    **for** i=0 **to** *n*       $F_{i}=f_{0},f_{1},\ldots ,f_{p}$ ***p* features in Image**       $D\leftarrow F_{i}$    **for** $f_{k}\in D$       **if** $\left(f_{k}\right)\in F_{n}\mathrm{and}\left(f_{k}\right)\notin U_{n}$ **then**           **if** $count(f_{k})=n$ **then**              $R\leftarrow (f_{k})$           **end**       **end**    **end**

**Figure 10:**
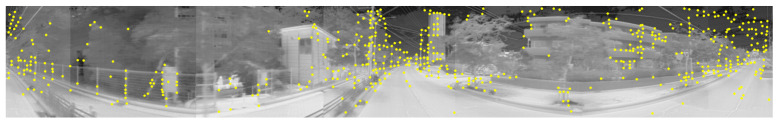
Visualization of features extracted from thermal images.

The same extraction algorithm is applied to thermal images; however, the semantic segmentation-based noise filtering will not be as effective as when applied to RGB images. The most important reason is the lack of a dataset for panoramic images. Meanwhile, the current prominent semantic segmentation algorithms for thermal images, such as PSTNet [12], AFNet [51], ABMDRNet [52], MMNet [53], FuseSeg [54], and MFNet [55], require a dataset in which RGB and thermal images have been calibrated, and then the results from RGB images will be used to train thermal images. Using datasets from cameras with narrow fields of view can affect the segmentation results of the panorama images. To handle this, the key features will still be extracted first as Figure 10. Then, during the calibration of RGB–thermal images, the outlier pairs will be filtered. This method will be presented in Section 3.2 of the paper.

#### 3.1.2. Feature Extraction from LiDAR Point Clouds

**Figure 11:**
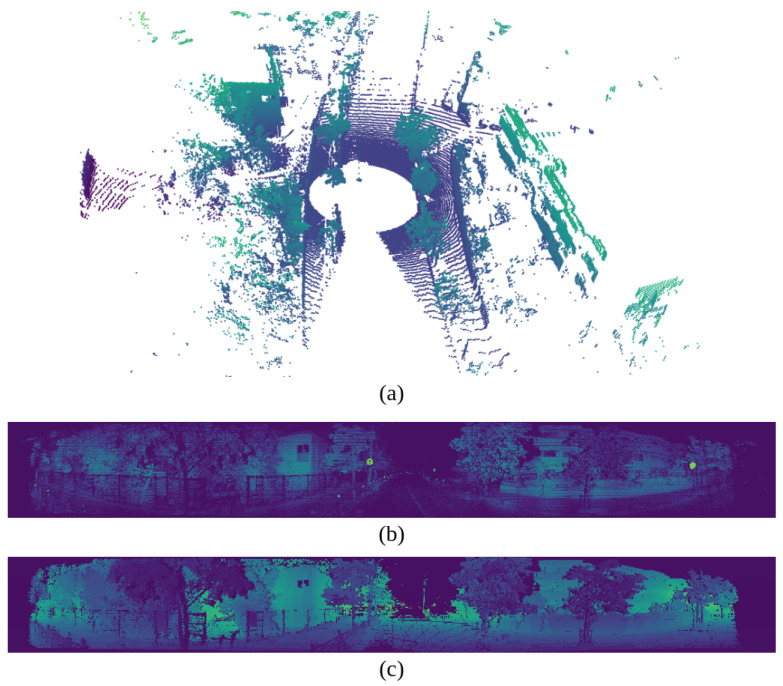
Visualization of image projection. (**a**) shows the 3D point cloud data from the LiDAR. (**b**) presents the 2D image data with the intensity channel. (**c**) presents the 2D image data with the range channel. The height of the image is 128, corresponding to the number of channels in the LiDAR.

After the reliable features in RGB images are extracted, these features are transformed to LiDAR images to identify reliable features on this dataset. Due to the lack of LiDAR datasets, the target calibration method will be used here to build a suitable feature extraction dataset. To avoid the impact of motion on the shape of objects in images and point clouds, the frames selected for training will have low speed. A crucial issue to address when converting from 3D point clouds to 2D images, as in Figure 11, is to minimize information loss of the points. This paper employs Spherical Projection to create a 360-degree panoramic image that encompasses the entire environment around the sensors. The projection algorithm first converts the coordinates in 3D space by transforming the Cartesian coordinates (x,y,z) of a point into spherical coordinates (ϕ,θ). ϕ in Equation (1) is the azimuth angle, which is the horizontal angle relative to the *x* axis, while θ in Equation (2) is the zenith angle, which is the vertical angle relative to the *z* axis.

(1) ϕ=arctan2(y,x),0<ϕ<π

(2) $$\theta =\arcsin(\frac{z}{\sqrt{x^{2}+y^{2}+z^{2}}}),\;0<\theta <2\pi$$

Then, the algorithm projects these coordinates onto a 2D plane with coordinates (u,v). *W* and *H* in Equation (3) are the width and height of the 2D image, respectively, with the product of *W* and *H* being the total number of points in one frame. This method helps reduce distortion near the sensors, which facilitates image processing by ensuring that key points close to the sensors are preserved more accurately [56].

(3) $$u=W\left(\frac{\phi }{2\pi }\right)\;\mathrm{and}\;v=H\left(\frac{\theta }{\pi }\right)$$

**Figure 12:**
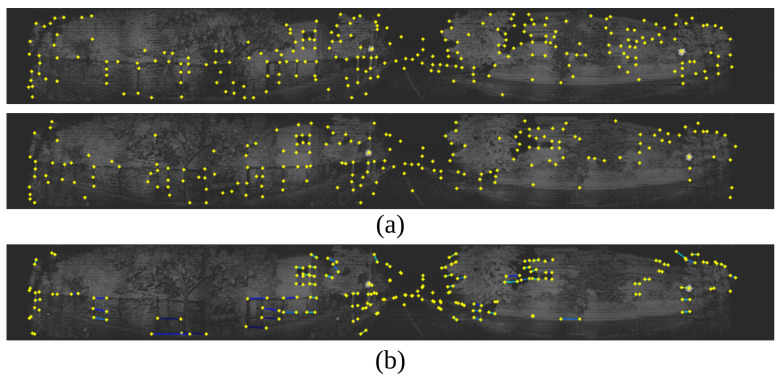
Visualization of key points extracted from LiDAR images. (**a**) simulates key points across two frames, while (**b**) simulates selecting key points with similarity across the two frames.

**Figure 13:**
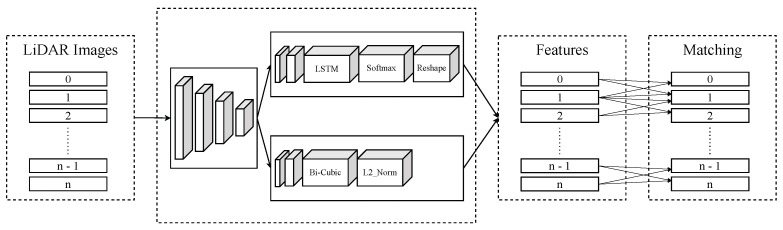
Pipeline of our approach. Key features of projected images are extracted by Superpoint enhanced by LSTM. These features are matched to find pair points in two consecutive frames.

We also enhance the SuperPoint algorithm by LSTM [57] to assess the similarity of key points, facilitating the matching of images across consecutive frames. From these points, the Brute-Force matcher is employed to detect corresponding point pairs across the frames, as in Figure 12. The method is presented in Algorithm 2. The Input will consist of images that have been converted from 3D point clouds to 2D images. Features will be extracted from these images and put in two sets $F_{i}$, $F_{i+1}$. If a feature appears in both images, that feature will be selected. The process for matching is presented in Figure 13. $f_{t}$ in Equation (4) determines information of features being related from the previous hidden state.

(4) $$f_{t}=\sigma \left(W_{f}\left[h_{t-1},x_{t}\right]+b_{f}\right)$$

Cross-Entropy Loss Function of Interest Point Decoder:

(5) $$L_{I}=\frac{1}{HW}\sum_{h=1,w=1}^{H,W}log\left(f{\left(x\right)}_{hw}\right)$$

We use Correspondence Contrastive Loss to encourage the network to learn descriptors. $s_{hw}=1$ for a positive pair and $s_{hw}=0$ for a negative pair.

(6) $$\begin{array}{c} L_{D}=\frac{1}{HW}\sum_{h=1,w=1}^{H,W}\left[s_{hw}{||{g\left(x\right)}_{hw}-g^{\prime }{\left(x\right)}_{hw}||}^{2}+\left(1-s_{hw}\right)max(0,m-{||g{\left(x\right)}_{hw}-g^{\prime }{\left(x\right)}_{hw}||}^{2})\right] \end{array}$$

The Loss Function combines Interest Point and Descriptor. $L_{I}$ for Loss Function of Interest Point of the source images and $L_{I}^{\prime }$ Loss Function of Interest Point of the target images. We use the same α weight for two Loss Functions because it maintains the balance and influence between the two images. Since both the source and target images have equal importance in the feature matching process, if one image is assigned a higher weight, the model can focus more on that image and ignore information from the other image.

(7) $$L=\alpha L_{I}+\alpha L_{I}^{\prime }+\beta L_{D}$$

**Algorithm 2** Feature extraction in LiDAR images

INPUT
    **Images** $L_{0},L_{1},\ldots ,L_{n}$
RESULTS
    **for** i=0 **to** *n*       R←{∅}       D←{∅}       $F_{i}=f_{0},f_{1},\ldots ,f_{p}$ ***p* features in Image *i***       $F_{i+1}=f_{0},f_{1},\ldots ,f_{q}$ ***q* features in Image *i* + 1**       $D\leftarrow F_{i},F_{i+1}$       **for** $f_{k}\in D$           **if** $\left(f_{k}\right)\in F_{i}\cap F_{i+1}$ **then**              $R\leftarrow (f_{k})$           **end**       **end**    **end**


### 3.2. Registration

After correcting the distortions in the point clouds caused by dynamic situations, RGB–LiDAR images and RGB–thermal images are calibrated. With thermal images, we use the same algorithms for extract features and remove unreliable points as RGB images. There are two steps for matching pairs of points which are rough matching and fine matching.

#### 3.2.1. Coarse Matching

After extracting features on LiDAR images, RGB images and thermal images we use Euclidean distance, as in Equation (8), and a histogram of oriented gradients [58], as in Equation (9), to match features.

(8) $$\sqrt{{\left(v_{ax}-l_{bx}\right)}^{2}+{\left(v_{ay}-l_{by}\right)}^{2}}\leq \Delta \left(x,y\right)$$

(9) $$arctan\left(\frac{G_{vy}}{G_{vx}}\right)-arctan\left(\frac{G_{iy}}{G_{ix}}\right)\leq \Delta \theta$$

#### 3.2.2. Fine Matching

Once the matching point pairs are identified, outlier pairs are filtered using the RANSAC algorithm [59] with Mahalanobis distance [60], as in Equation (10). *Q* is the set of features, $\vec{x}$ is the vector of features, $\vec{\mu }$ is the mean vector of the set, and *S* is the covariance matrix of the set.

(10) $$d_{M}\left(\vec{x},Q\right)=\sqrt{{\left(\vec{x}-\vec{\mu }\right)}^{T}S^{-1}\left(\vec{x}-\vec{\mu }\right)}$$

## 4. Ego-Motion Compensation

This section focuses on ego-motion compensation, where we present a comprehensive compensation method for both camera and LiDAR data, achieved through point accumulation. The findings in this section contribute to improving calibration accuracy.

### 4.1. Point Cloud Accumulation

**Figure 14:**
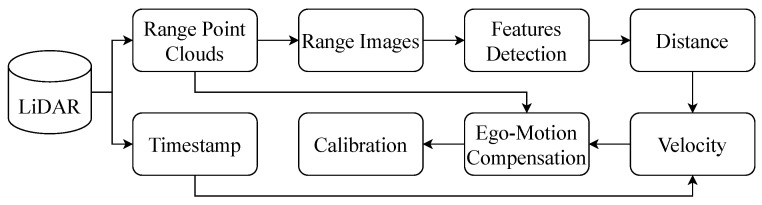
Pipeline of the ego-motion compensation process. First, the point clouds are converted into two-dimensional images using Spherical Projection. Key features are then identified within these range images, and corresponding point pairs are matched. By matching key feature pairs, the distance between frames can be determined, allowing for velocity estimation. Finally, velocity and timestamp will be used to resolve ego-motion compensation and point cloud accumulation.

The first step in the process is to estimate the velocity of the vehicle. Any inaccuracies in estimating the vehicle’s velocity can lead to errors in processing dynamic distortion. To estimate velocity solely based on the point cloud data, we must determine information regarding the distance traveled and the time taken. While the time can be extracted from the LiDAR timestamps, determining the distance traveled is a more complex issue. A common method for distance determination using LiDARs is to identify significant features in the frames and then derive the transformation matrix for these frames. The general steps of the method are described in Figure 14. Our method contributes not only the necessary step for targetless calibration in moving situations, but also a novel way for points accumulation research without support from other sensors. With conventional algorithms, the time and computational resources required to match all the points in a 3D frame can be highly significant. This is because the process of matching each point in a dense 3D point cloud involves complex calculations. However, a more efficient approach can be achieved by reducing the calculations needed for matching. This is performed by projecting the 3D points into 2D images and selecting the most relevant or similar point pairs across different frames in 2D space. Once these point pairs are identified in 2D, they are re-projected back into 3D space, where their coordinates are used to calculate the transformation matrix. This transformation matrix describes the geometric relationship between the two frames, allowing for efficient alignment without needing to match every point in the original 3D dataset. Using the identified point pairs, we apply Singular Value Decomposition (SVD) [61,62] to determine the transformation matrix between the point clouds. SVD is a numerically stable method that avoids problems caused by degenerate matrices or bad condition matrices during computation.

We have two sets of points $\left\{\left(k_{j}^{P_{i}},k_{j}^{P_{i+1}}\right)|j=1,2,\ldots ,m\right\}$, where $\left(k_{j}^{P_{i}},k_{j}^{P_{i+1}}\right)$ represents a pair of corresponding points between the two sets as Equation (11).

(11) $${\bar{k}}^{P_{i}}=\frac{1}{n}\sum_{j=1}^{n}k_{j}^{P_{i}}\;\mathrm{and}\;{\bar{k}}^{P_{i+1}}=\frac{1}{n}\sum_{j=1}^{n}k_{j}^{P_{i+1}}$$

The matrix *D* in Equation (12) represents the covariance between two sets of points. It calculates the difference between each point and the centroid in both sets, then multiplies these differences to form a covariance matrix.

(12) $$D=\sum_{j=1}^{n}\left(k_{j}^{P_{i+1}}-{\bar{k}}^{P_{i+1}}\right){\left(k_{j}^{P_{i}}-{\bar{k}}^{P_{i}}\right)}^{T}$$

In Equation (13), *U* and *V* are orthogonal matrices, and Σ is a diagonal matrix of singular values. The SVD allows us to extract the rotation and alignment information between the two sets of points.

(13) $$U\Sigma V^{T}=D$$

*R* is the rotation matrix that describes how the points are rotated, sets and *d* is the translation vector, which describes the shift between the centroids of the two point sets after accounting for rotation in Equation (14). These provide the necessary information to fully define the spatial transformation between the two sets of 3D points.

(14) $$R=VU^{T}\;\mathrm{and}\;d={\bar{k}}^{P_{i+1}}-VU^{T}{\bar{k}}^{P_{i}}$$

Finally, *M* is the homogeneous transformation matrix. The matrix provides a complete description of the transformation needed to move from the reference frame of one set of points $P_{i}$ to the reference frame of the other set of points $P_{i+1}$. This transformation matrix is essential in applications such as 3D point cloud alignment, robotics, and computer vision for determining how objects or scenes have shifted between two frames. Equation (15) is subsequently applied to the entire point cloud to calculate the distances between frames.

(15) $$M=\left[\begin{array}{cc} R & d\\ 0 & 1 \end{array}\right]$$

### 4.2. Ego-Motion Compensation in LiDARs

**Figure 15:**
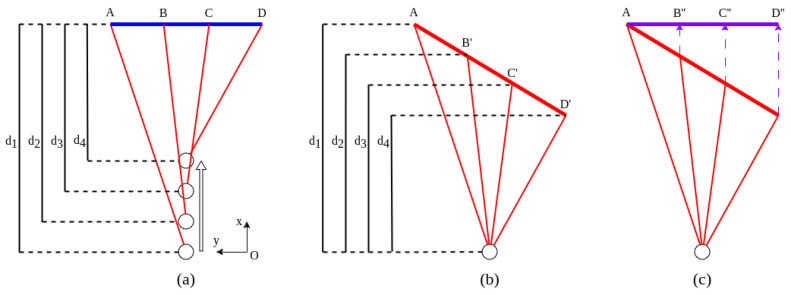
Visualization of distortion correction. The motion of the vehicle is presented by the circles, and the LiDAR is also sotating while the vehicle is in motion. (**a**) shows the actual shape of the obstacle. (**b**) depicts the shape of the obstacle scanned by LiDAR. (**c**) illustrates the shape of the obstacle after distortion correction.

Figure 15 shows the distortion correction process for the mechanical 3D LiDAR, specifically, and rotating LiDARs in general. In this case, the LiDAR scans counter clockwise (from left to right); AD represents the actual shape of the obstacle, with the vehicle moving closer to the obstacle, as shown in Figure 15a. When this sensor moves forward, at four different positions, the LiDAR records four different distance values, respectively $d_{1}$, $d_{2}$, $d_{3}$, and $d_{4}$. Consequently, the shape captured by the LiDAR appears as $AD^{\prime }$ in Figure 15b. To obtain the actual shape as AD, a transformation is required to convert points $B^{\prime }$, $C^{\prime }$, and $D^{\prime }$ into points $B^{\prime \prime }$, $C^{\prime \prime }$, and $D^{\prime \prime }$, as in Figure 15c.

With the estimated velocity $v_{i}$ and $\Delta t_{l}$ of $k_{l}^{P_{i}}$ in the $P_{i}$ set, the points in the point clouds can be corrected. The rotation speed of the LiDAR is $\omega_{i}$, and $\hat{\omega_{i}}$ is the skew-symmetric matrix which is presented in Equation (16). The parameters $\sin(\alpha_{l})$ and $\cos(\alpha_{l})$ are trigonometric functions that control the amount of rotation.

(16) $$R_{l}=I+\sin\left(\alpha_{l}\right)\hat{\omega_{i}}+\left(1-\cos\left(\alpha_{l}\right)\right){\hat{\omega_{i}}}^{2}\;\mathrm{with}\;\alpha_{l}=\omega_{i}\Delta t_{l}$$

Equation (17) describes the translation vector $d_{l}$, representing how far the point has moved during the time $\Delta t_{l}$.

(17) $$d_{l}=v_{i}\Delta t_{l}=d\frac{\Delta t_{l}}{\Delta t_{i}}$$

Equation (18) corrects the distortion of the point by applying both rotation and translation, adjusting the point’s position based on the ego-motion of the sensor

(18) $$k_{l}^{\prime P_{i}}=R_{l}k_{l}^{P_{i}}-d_{l}$$

**Figure 16:**
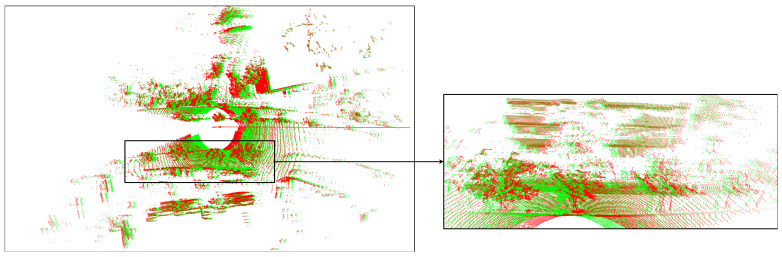
Visualization of the differences in distortion correction on 3D point clouds within a frame with a speed of 54 km/h and a frequency of 10 Hz. The red part shows the original points of the point clouds, while the green part shows the corrected points. The left image shows points on the xy-plane. The right image shows points on the yz-plane.

The result of the ego-motion compensation of LiDAR point clouds is shown in Figure 16. With middle or high speed, the difference in position of points is clear. In general, the algorithm for velocity estimation is presented in Algorithm 3. The input to the algorithm will be LiDAR images $I_{i}$ projected from point clouds $P_{i}$. These images include keypoints $K_{i}$. The keypoints on two consecutive LiDAR images $k_{j}^{I_{i}}$ are calculated to match the points for the 2D image. After finding these points, points are converted to the original 3D space $k_{j}^{P_{i}}$. From these point pairs, the velocity $v_{i},\omega_{i}$ and *M* are calculated between the source point sets and the target point sets. The matrix *M* then is used to transform all points in $P_{i+1}$ to process ego-motion compensation. The processed point $P_{i+1}^{\prime }$ is put into a set *D* with the processed point clouds.
**Algorithm 3** Velocity estimationINPUT    **Point Clouds** $P_{1},P_{2},\ldots ,P_{n}$    **Projected Images** $I_{1},I_{2},\ldots ,I_{n}$    **Timestamps** $T_{1},T_{2},\ldots ,T_{n}$    **Keypoints** $K_{1},K_{2},\ldots ,K_{n}$    **Point accumulation** D=⌀RESULTS    **for** i=1 **to** *n* − 1       M=[⌀]       $\Delta t_{i}=T_{i+1}-T_{i}$       $\left(k_{j}^{I_{i}},k_{j}^{I_{i+1}}\right)|j=1,2,\ldots ,m\leftarrow \left(K_{i},K_{i+1}\right)$       $\left(k_{j}^{P_{i}},k_{j}^{P_{i+1}}\right)\left|j=1,2,\ldots ,m\leftarrow \left(k_{j}^{I_{i}},k_{j}^{I_{i+1}}\right)\right|j=1,2,\ldots ,m$       $d_{i},R_{i}\leftarrow \left(k_{j}^{P_{i}},k_{j}^{P_{i+1}}\right)|j=1,2,\ldots ,m$       $v_{i}=d_{i}/\Delta t_{i}$       $\omega_{i}=2\pi /\Delta t_{i}$       $M\leftarrow (v_{i},\omega_{i})$       $P_{i+1}^{\prime }\leftarrow M\left(P_{i+1}\right)$       $D\leftarrow P⋃P_{i+1}^{\prime }$    **end**

### 4.3. Ego-Motion Compensation in Cameras

**Figure 17:**
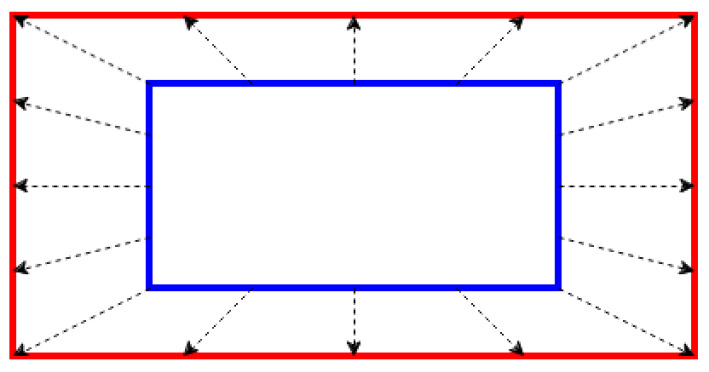
Visualization of distortion correction of cameras. The blue rectangle is the actual shape, and the red rectangle is the shape distorted by ego-motion.

While 3D ego-motion compensation delivers highly accurate information regarding distances and the 3D structure of the environment for LiDAR sensors, 2D ego-motion compensation for cameras operates within the two-dimensional space of the image as Figure 17. This approach focuses on adjusting the image data to account for optical flow in the 2D images and plays a crucial role in calibration. Even small changes can influence how pixels shift and are analyzed throughout a sequence of images, potentially impacting the overall accuracy and quality of image processing and calibration. We obtain the new positions of pixels based on brightness constancy constraint [63] as Equation (19).

(19) $$I\left(x_{2D},y_{2D},t\right)=I\left(x_{2D}+\Delta x_{2D},y_{2D}+\Delta y_{2D},t+\Delta t\right)$$

The changes in images are calculated based on the perspective projection as Equation (20) with $f_{x},f_{y}$ being the focal length, and $c_{x},c_{y}$ being the principal point. *R* is rotation of the camera and *T* is translation of the camera.

(20) $$\left[\begin{array}{c} \Delta x_{2D}\\ \Delta y_{2D}\\ 1 \end{array}\right]=K\left[\begin{array}{c} R|T \end{array}\right]\left[\begin{array}{c} \Delta x_{3D}\\ \Delta y_{3D}\\ \Delta z_{3D}\\ 1 \end{array}\right]=\left[\begin{array}{cccc} f_{x} & 0 & c_{x} & 0\\ 0 & f_{y} & c_{y} & 0\\ 0 & 0 & 1 & 0 \end{array}\right]\left[\begin{array}{cc} R & T\\ 0 & 1 \end{array}\right]\left[\begin{array}{c} v_{3D}\Delta t\\ v_{3D}\Delta t\\ v_{3D}\Delta t\\ 1 \end{array}\right]$$

**Figure 18:**
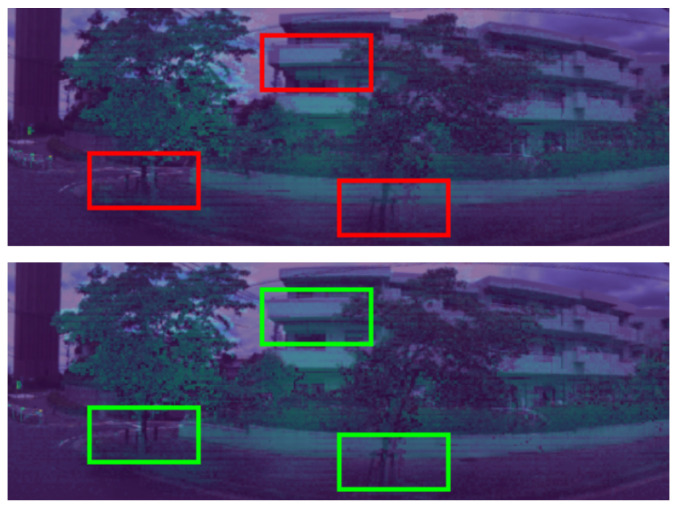
Visualization of 360 RGB–LiDAR images calibration. The (**above image**) including the red rectangles indicate the calibration results before applying correction. The (**below image**) including the green rectangles indicates the calibration results after applying correction.

The RGB–LiDAR image registered results are presented in Figure 18.

**Figure 19:**
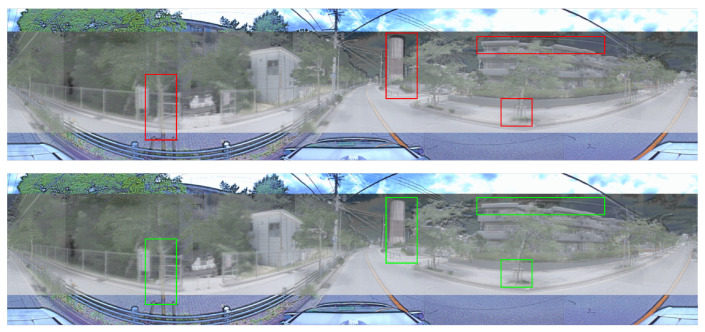
Visualization of 360 RGB–thermal images calibration. The (**above image**) includes red rectangles that indicate the calibration results before applying correction. The (**below image**) includes green rectangles that indicate the calibration results after applying correction.

The RGB–thermal image registered results are presented in Figure 19.

## 5. Experiment Results

This section provides the experimental results, where we address three main objectives: first, validating the reliability of the velocity estimation method through point accumulation using various LiDAR models; second, comparing our calibration approach to others for 360 RGB and thermal cameras; and third, analyzing calibration outcomes for 360 RGB cameras with LiDARs in both static and dynamic scenarios, with dynamic tests conducted at multiple speed levels. The effectiveness of ego-motion compensation may vary across cases, and we further compare our findings to established methodologies.

**Figure 20:**
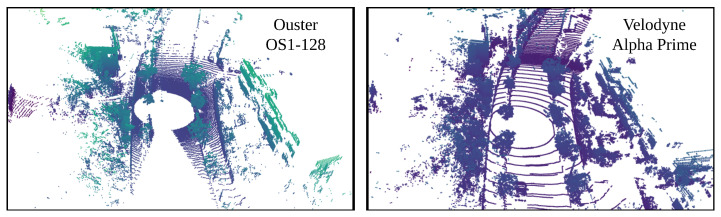
Visualization of point clouds extracted from Ouster OS1-128 and Velodyne Alpha prime.

To evaluate the performance of our method, we use a dataset collected around Nagoya University, Japan. The dataset was collected using three types of sensors: 360 RGB images, 360 thermal images, and LiDAR point clouds. The 360 RGB images were captured from five LadyBug cameras with a 90-degree field of view each. The 360 thermal images were captured from six FLIR ADK cameras, each with a 75-degree field of view. The LiDAR used in this study is the Ouster OS1-128 and Velodyne Alpha Prime. These are two mechanical LiDAR sensors designed to deliver high-resolution data at a scanning frequency of 10 Hz. Both LiDARs provide detailed and extensive information, including range and intensity data. This high data acquisition rate enables the generation of detailed and consistent point clouds, essential for accurate spatial analysis. Both LiDARs have 128 channels, but their channel distributions are different. While the Ouster has nearly uniform channel spacing, the Velodyne features wider spacing between the first and last channels compared to the middle channels as Figure 20.

**Figure 21:**
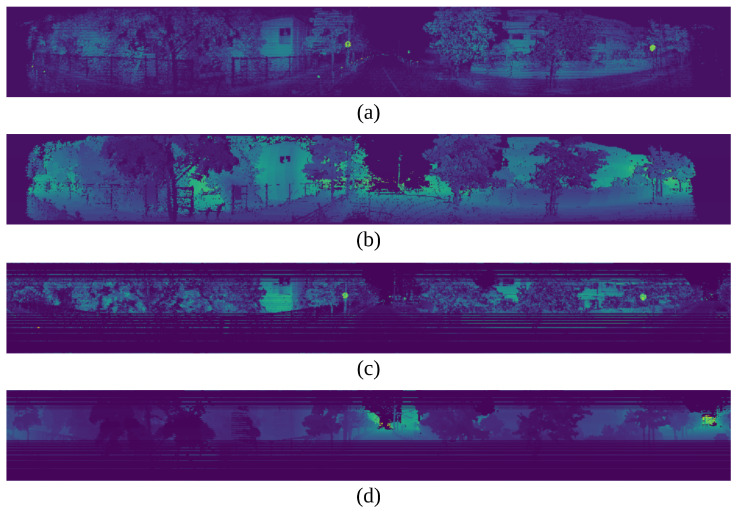
Visualization of image projection. (**a**) presents the 2D image data with the intensity channel from Ouster OS1-128. (**b**) presents the 2D image data with the range channel from Ouster OS1-128. (**c**) presents the 2D image data with the intensity channel from Velodyne Alpha prime. (**d**) presents the 2D image data with the range channel from Velodyne Alpha prime.

Both distributions have their advantages and disadvantages. The Velodyne’s distribution concentrates points densely in the middle channels, making it easier to recognize objects at a distance. On the other hand, the Ouster’s uniform distribution helps create LiDAR images that resemble RGB or thermal images as Figure 21.

Our dataset includes both static and dynamic scenarios, offering a diverse range of conditions for testing and validating different sensor technologies. The data are corrected from actual speeds taken directly from the Control Area Network (CAN) bus data. We evaluate our method based on the estimated velocity results and the targetless calibration results. With the targetless calibration results, we evaluate three cases including stopping, going straight and turning left or right. Calibration results are compared based on multiple speed ranges.

### 5.1. Velocity Estimation

**Figure 22:**
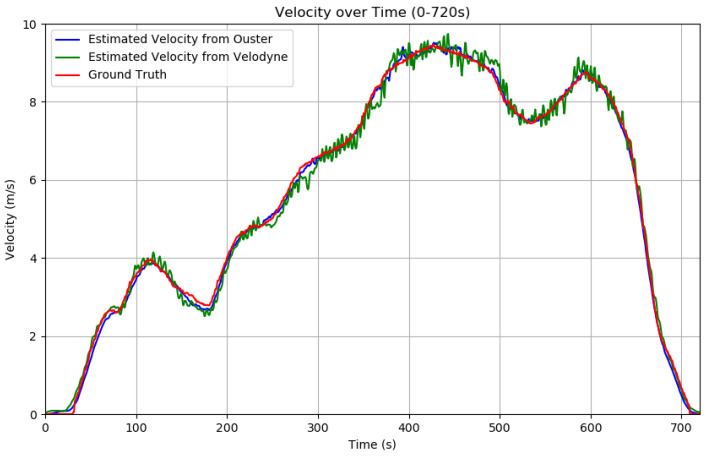
Visualization of velocity comparison between estimated velocity and ground truth over a continuous duration of 720 s. The intervals between approximately 100 to 200 s and 400 to 500 s corresponded to periods when the vehicle was turning. Conversely, the intervals from 0 to approximately 100 s, 200 to 400 s, and 500 to 600 s represented phases when the vehicle was moving straight. The vehicle decelerated and came to a halt between 600 and 720 s. The maximum observed velocity difference was 0.36 m/s, while the average velocity difference over the 720 s period was 0.03 m/s, as in Table 1.

Figure 22 illustrates a comparative analysis between the actual speed data and the estimated speed data obtained by matching key points between two consecutive frames. The analysis was carried out under rainy weather conditions to evaluate the performance of the algorithm in environments that might influence LiDAR accuracy [4]. Material properties can also lead to errors of LiDAR measurements [64]. To address these potential issues and prevent velocity measurements from becoming disproportionately large or small, a moving average technique [65] with a window size of 3 was applied. The results of the analysis reveal that the velocity estimation algorithm, leveraging key points from consecutive frames, proves both efficient and resilient, making it ideal for applications like sensor calibration. Its ability to maintain high accuracy under fluctuating conditions highlights its practical value in real-world scenarios, where environmental factors may otherwise compromise measurement precision. Nevertheless, differences emerge when comparing data from the Velodyne Alpha Prime and Ouster OS1-128 sensors. While Velodyne’s estimated mean values are closer to the ground truth, they exhibit great variability, suggesting that Velodyne’s outputs are less consistent than those from Ouster. This discrepancy is largely due to the distributions of channels. Ouster’s evenly spaced channels allow for better feature identification in close proximity. With Velodyne’s channels, extracting features from distant objects poses significant challenges due to their diminished size, which results in a reduced level of detail. Consequently, this leads to more pronounced deviations in point matching when compared to Ouster’s data. The above results show that the efficiency of the algorithm will depend on the type of LiDARs.

### 5.2. 360 RGB Camera–360 Thermal Camera Registration

We evaluate our method with CNN [66], RI-MFM [67], and RIFT [68] based on Mean Absolute Error (MAE) [69], Root Mean Square Error (RMSE) [70], and Accuracy. We also evaluate with our method after applying ego-motion compensation. While MAE is a measure of the average absolute difference between predicted and actual data, RMSE calculates the average deviation, expressed in the same units as the original data, between predicted and actual values.

(21) $$MAE=\frac{1}{n}\sum_{i=1}^{n}\left|q_{i}-q_{i}^{T}\right|$$

(22) $$RMSE=\sqrt{\frac{1}{n}\sum_{i=1}^{n}{\left(q_{i}-q_{i}^{T}\right)}^{2}}$$

Accuracy is the ratio of correctly classified points to the total number of points.

(23) $$Accuracy=\frac{NumberofCorrectPredictions}{TotalNumberofPredictions}$$

We use the Manhattan Distance [71] to set a threshold for points considered correct.

(24) $$|x-\bar{x}|+|y-\bar{y}|\leq \Delta \left(x,y\right)$$

**Figure 23:**
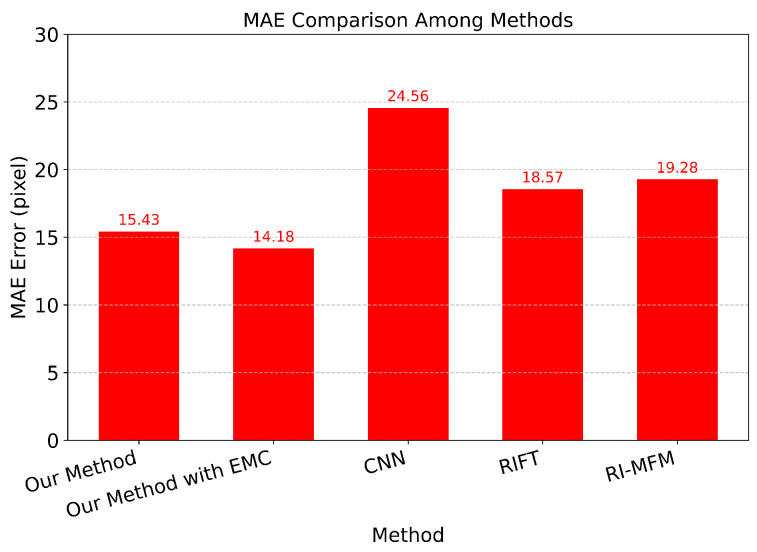
Comparison with CNN, RIFT, RI-MFM by MAE.

**Figure 24:**
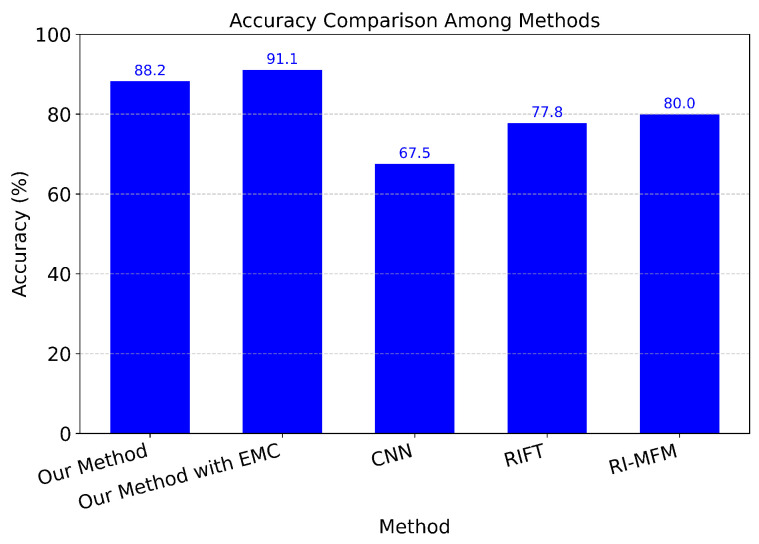
Comparison with CNN, RIFT, RI-MFM by Accuracy.

Figure 23 and Figure 24 present the results of the MAE and Accuracy comparisons. The data of the MAE comparison demonstrate that, although employing the same feature extraction techniques as RIFT and RI-MFM across two distinct image types, our method achieves higher performance in terms of the total number of features identified. This enhanced performance is attributed to our approach’s ability to account for differences in illumination when matching point pairs between cameras, whereas other extraction methods apply a uniform illumination assumption across the entire thermal image. With the Accuracy metric, we set the Manhattan Distance threshold to be less than 20 in order to be considered an accurate score. The results indicate that not only does the average distance yield the smallest error, but the percentage of correctly matched scores is also the highest.

**Figure 25:**
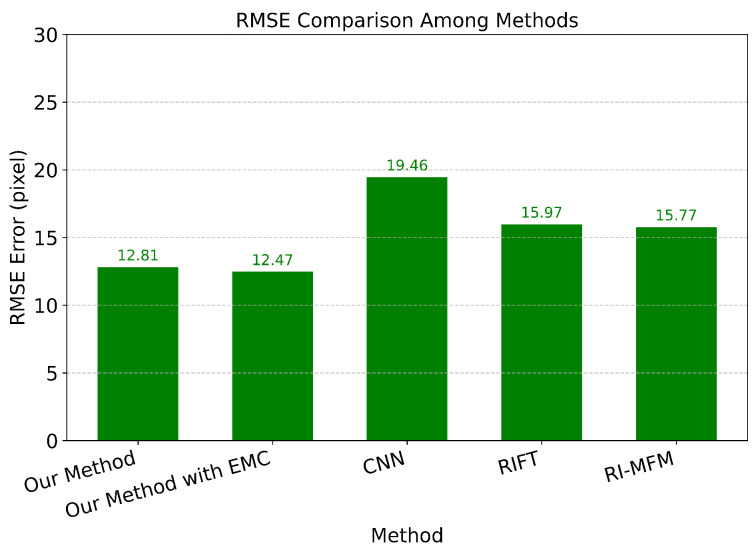
Comparison with CNN, RIFT, RI-MFM by RMSE.

Additionally, we conduct an RMSE comparison, as illustrated in Figure 25, where our method demonstrates better performance. RMSE emphasizes the impact of larger errors, making it particularly sensitive to outliers. Achieving a strong result in RMSE comparison suggests that the model not only has high accuracy, but also effectively manages both typical values and those that deviate significantly. When both MAE and RMSE scores are favorable, it implies that the model consistently fits the data while also accommodating fluctuations and outliers, maintaining prediction errors within acceptable limits. This reflects the model’s capacity to produce reliable predictions across the entire range of data, enhancing its robustness and overall predictive reliability.

Unlike the aforementioned methods, our technique effectively eliminates most noisy points while retaining only the essential points. This selection process ensures that the chosen point pairs are more accurate than those derived from extracting the maximum number of features. Our method after applying ego-motion compensation has slightly better results. The reason for the difference is that the focal length and shutter speed parameters of both cameras are different, which leads to the position of the extracted points changing. Despite the improvement in results, the difference is not too large with an accuracy increase of 1.9 percent, a MAE error decrease of 1.25, and a RMSE error decrease of 0.34. However, in applications that require high accuracy such as object recognition at high velocities, this difference can have a big impact on vehicle safety.

### 5.3. 360 RGB Camera–LiDAR Registration

#### 5.3.1. Quantitative Results in Static Situations

With LiDAR and 360 RGB camera, we evaluate by rotations $(\theta_{x},\theta_{y},\theta_{z}$) and translations $\left(t_{x},t_{y},t_{z}\right)$, respectively.

**Figure 26:**
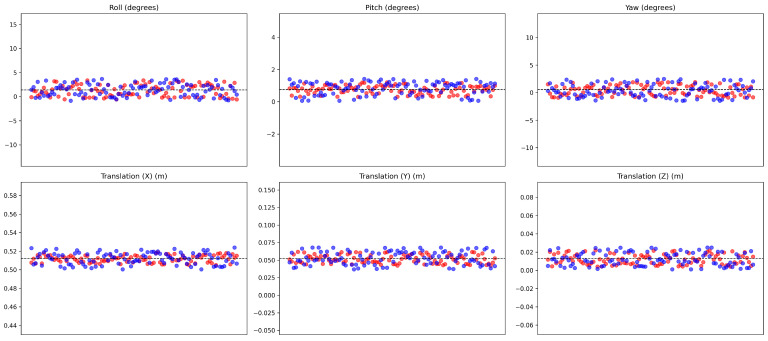
Red points represent the results of calibration without distortion correction, while blue points represent the results with distortion correction in static situations. The dashed line is the results from the target based method.

Figure 26 and Table 2 show the results of the samples in static situations. The results were tested over 100 samples, and Table 2 describes the deviations of Rotation in degrees and Translation in meters. The data show that there is not much difference between the targetless calibration algorithm with and without distortion correction, nor when compared to the results obtained with target calibration. According to Table 2, the targetless calibration algorithm without distortion correction gives slightly better results than with distortion correction in both Rotation and Translation. This may be due to the fact that, although the device is stationary, small variations still appear between LiDAR scans, just like the problems in velocity calculation. These small deviations lead to differences in distance measurements between consecutive scans, causing errors in the calibration process. Compared to the target calibration method, differences still occur in both methods and with both the Ouster and Velodyne LiDARs.

#### 5.3.2. Quantitative Results in Dynamic Situations

**Figure 27:**
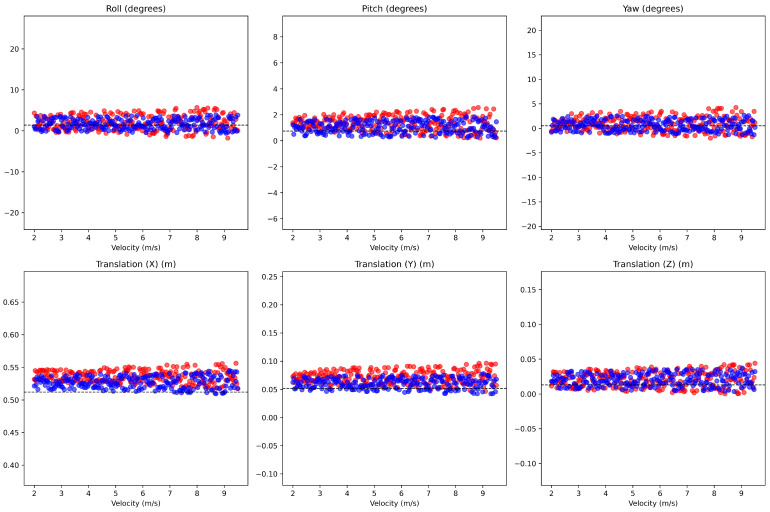
Red points and blue points represent the results of calibration without and with distortion correction in dynamic situations. The dashed lines present the results using the actual data.

In dynamic scenarios, the differences are more pronounced, as shown in Figure 27 and Table 3. The dataset in this experiment consists of 250 samples in the speed range from 2 m/s to 9.5 m/s, which have been filtered for outliers through the RANSAC algorithm. Table 3 describes the deviations of Rotation in degrees and Translation in meters. When the speed is below a certain threshold, the variation is still relatively small, and may not significantly affect actual operation if ego-motion compensation is not applied. This suggests that distortion correction can be ignored during operation, provided that the equipment and vehicle maintain low speeds subject to specific constraints. However, when the speed exceeds 4 m/s, the calibration errors due to not applying ego-motion compensation begin to become more apparent. When distortion correction is applied, it ensures that the calibration remains consistent even at higher speeds, where rapid motion can degrade the accuracy of the sensor fusion process. Especially with Translation, although there are fluctuations in individual values, the average value is generally maintained stable.

#### 5.3.3. Comparison

**Figure 28:**
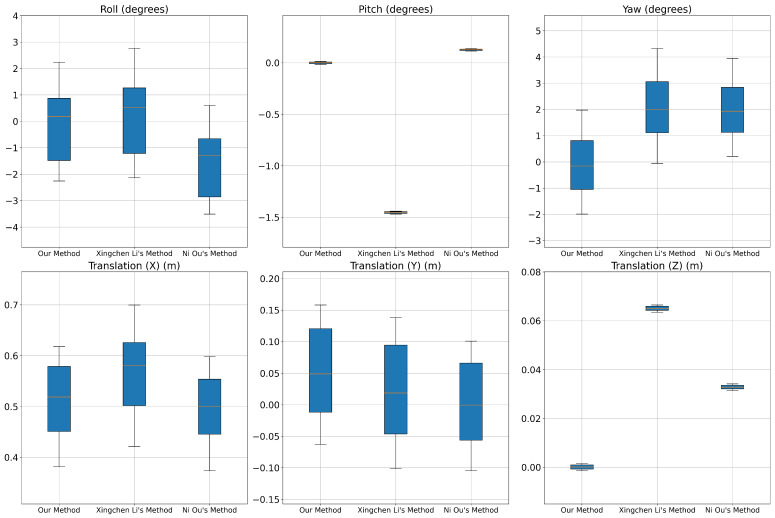
Comparison of error in rotation and translation of three methods.

We conduct an in-depth comparative analysis of our method against the techniques proposed by Ni Ou [72] and Xingchen Li [73], ensuring objectivity and comprehensiveness by applying them to the same real-world dataset. As illustrated in Figure 28, this analysis encompasses various scenarios, from static to dynamic environments and at different speeds. Ni Ou’s method relies on aligning the LiDAR point cloud with projected points from images, while Xingchen Li’s approach emphasizes edge extraction from both LiDAR and image data. The ground truth values for the parameters Roll, Pitch, Yaw, X, Y, and Z are 1.3757°, 0.7452°, 0.5159°, 0.512 m, 0.052 m, and 0.013 m, respectively.

Regarding rotation parameters, our method shows significantly higher stability. Specifically, the Roll parameter has a mean of −0.1053° and a median of 0.1837°. In contrast, Xingchen Li’s method has a mean of 0.2339° and a median of 0.5269°. Notably, Ni Ou’s method shows a mean of −1.5520° and a median of −1.2831°. While the mean values of Xingchen Li’s method are better, the higher number of outliers indicates that edge extraction can significantly impact the results across different scenarios. For the Pitch parameter, our method and Ni Ou’s method achieves mean and median values close to 0.7452°, significantly greater than Xingchen Li’s method. For Yaw, our method has a mean of −0.0603° and a median of −0.0027°, which are better than Xingchen Li’s method has values of 2.1102° and 1.9985°, and Ni Ou’s method has values of 2.0222° and 1.9328°.

Regarding translation parameters, on the *x*-axis, our method has a mean of 0.5336 m and a median of 0.5259 m. In contrast, Xingchen Li’s method shows higher mean and median values with 0.5651 m and 0.5812 m, respectively, indicating greater instability. Ni Ou’s *x*-axis results are lower, with a mean of 0.4976 m and a median of 0.5008 m which is lower than our values. The reason for this discrepancy is primarily that our algorithm maintains a high density of points distributed along the *x*-axis, leading to potential misalignments when combining points if the specified distance is not sufficiently large. Conversely, while using the feature extraction, Ni Ou’s method significantly reduces the number of extracted points from the images.

Nonetheless, for the *y* and *z* axes, our method demonstrates better stability, with mean and median values closely aligned with the ground truth (0.0477 m and 0.0489 m for the *y* axis; 0.0008 m and 0.0005 m for the *z* axis). In comparison, Xingchen Li’s approach (0.0264 m and 0.0286 m for the *y* axis; 0.0650 m and 0.0650 m for the *z* axis) and Ni Ou’s method (−0.0010 m and −0.0005 m for the *y* axis; 0.0328 m and 0.0328 m for the *z* axis) exhibit noticeable fluctuations.

The results clearly indicate that our method yields values closer to the ground truth, especially in complex environments where edge-based techniques struggle due to noise and the loss of point clouds at object boundaries. This significant improvement highlights the strengths of our local feature strategy, which effectively filters out noise. However, the weaknesses observed in the *x*-axis require further attention.

## 6. Discussion

Despite the advancements outlined in this paper, several challenges still persist. A significant issue involves the accuracy of instantaneous velocity computation. Addressing outliers in the time intervals used can prove difficult, particularly when the environment contains infrared-absorbing objects or surfaces lacking reflectivity. These conditions hinder the ability of LiDAR systems to effectively match points, even when the vehicle remains stationary. Additionally, calibration under varying lighting conditions and adverse weather remains a concern. While the proposed calibration and distortion correction methods perform well in light rain, they have yet to be tested in more extreme conditions, such as snow, dust, or heavy rainfall, which can severely impair both the scanning capabilities of LiDAR and the imaging performance of cameras.

## 7. Conclusions

This paper introduces an innovative method for external calibration of multi-sensor systems, eliminating the need for traditional calibration objects while enabling efficient performance in dynamic environments. By integrating 360-degree RGB and thermal cameras, the approach effectively reduces blind spots, enhancing overall system performance in applications such as object detection and SLAM. Moreover, the combination of point extraction and ego-motion compensation for images and point clouds demonstrates substantial improvements over existing methods for calibrating RGB–LiDAR and RGB–thermal images. The variation in velocity influences the calibration outcomes between sensors. While the implementation of ego-motion compensation yields improved results under high-velocity conditions compared to cases without it, residual negative effects persist, ultimately diminishing overall calibration accuracy. In the near future, our research will prioritize refining the velocity calculation process, as improvements in instantaneous velocity estimation are especially beneficial when velocity measurement devices are unavailable. We also aim to expand our work to sensor calibration and integration, focusing on various cameras and LiDARs operating in challenging conditions like adverse weather. This will further improve the calibration process is practical accuracy, ultimately contributing to a more robust and adaptive system capable of tackling complex real-world scenarios. Furthermore, our objective is to sustain the stability of the proposed method under varying vehicle velocities, ensuring robust performance despite fluctuations in velocity. We will improve and develop the program to add this method to Autoware software [24], which is the biggest open-source project around the world for applications about cameras-LiDARs fusion and autonomous driving.

## Figures and Tables

**Table 1:** Maximum and average velocity measurements.

Name	Max Velocity	Mean Velocity
Ouster	9.51 m/s	5.56 m/s
Velodyne	9.74 m/s	5.59 m/s
Ground Truth	9.47 m/s	5.59 m/s

**Table 2:** Mean errors of targetless calibration in static situations.

Mean Errors	Without Ego-Motion Compensation	With Ego-Motion Compensation
Roll Error (degrees) (°)	1.0714	1.0886
Pitch Error (degrees) (°)	0.2162	0.3624
Yaw Error (degrees) (°)	0.7999	1.0015
Translation (X) Error (m)	0.0032	0.0054
Translation (Y) Error (m)	0.0051	0.0081
Translation (Z) Error (m)	0.0048	0.0064

**Table 3:** Mean errors of targetless calibration in dynamic situations.

Velocity	Mean Errors	Without Ego-Motion Compensation	With Ego-Motion Compensation
2 m/s–3 m/s	Roll Error (degrees) (°)	1.2446	1.1996
	Pitch Error (degrees) (°)	0.4448	0.3734
	Yaw Error (degrees) (°)	1.1874	1.1131
	Translation (X) Error (m)	0.0184	0.0121
	Translation (Y) Error (m)	0.0136	0.0086
	Translation (Z) Error (m)	0.0087	0.0080
3 m/s–4 m/s	Roll Error (degrees) (°)	1.3190	1.2146
	Pitch Error (degrees) (°)	0.5779	0.3440
	Yaw Error (degrees) (°)	1.2619	1.1733
	Translation (X) Error (m)	0.0268	0.0152
	Translation (Y) Error (m)	0.0185	0.0093
	Translation (Z) Error (m)	0.0099	0.0082
4 m/s–5 m/s	Roll Error (degrees) (°)	1.3797	1.2636
	Pitch Error (degrees) (°)	0.6200	0.3866
	Yaw Error (degrees) (°)	1.3021	1.1652
	Translation (X) Error (m)	0.0239	0.0137
	Translation (Y) Error (m)	0.0210	0.0102
	Translation (Z) Error (m)	0.0101	0.0088
5 m/s–6 m/s	Roll Error (degrees) (°)	1.4497	1.3053
	Pitch Error (degrees) (°)	0.7387	0.4228
	Yaw Error (degrees) (°)	1.3870	1.2093
	Translation (X) Error (m)	0.0213	0.0148
	Translation (Y) Error (m)	0.0206	0.0105
	Translation (Z) Error (m)	0.0110	0.0087
6 m/s–7 m/s	Roll Error (degrees) (°)	1.5521	1.2817
	Pitch Error (degrees) (°)	0.6482	0.4519
	Yaw Error (degrees) (°)	1.4743	1.2412
	Translation (X) Error (m)	0.0242	0.0157
	Translation (Y) Error (m)	0.0199	0.0108
	Translation (Z) Error (m)	0.0116	0.0094
7 m/s–8 m/s	Roll Error (degrees) (°)	1.6495	1.3175
	Pitch Error (degrees) (°)	0.7719	0.4841
	Yaw Error (degrees) (°)	1.6465	1.2775
	Translation (X) Error (m)	0.0236	0.0162
	Translation (Y) Error (m)	0.0189	0.0106
	Translation (Z) Error (m)	0.0132	0.0091
8 m/s–9 m/s	Roll Error (degrees) (°)	1.7663	1.3422
	Pitch Error (degrees) (°)	0.8377	0.5072
	Yaw Error (degrees) (°)	1.7007	1.2710
	Translation (X) Error (m)	0.0249	0.0154
	Translation (Y) Error (m)	0.0226	0.0114
	Translation (Z) Error (m)	0.0147	0.0110
9 m/s–9.5 m/s	Roll Error (degrees) (°)	1.9336	1.3637
	Pitch Error (degrees) (°)	0.9996	0.5446
	Yaw Error (degrees) (°)	1.8124	1.3053
	Translation (X) Error (m)	0.0256	0.0184
	Translation (Y) Error (m)	0.0241	0.0138
	Translation (Z) Error (m)	0.0153	0.0113

## Data Availability

The data presented in this study are available on request from the corresponding author.
